# Knowledge, attitudes, and practices in obesity among trained and in-training primary care providers in an urban safety-net hospital system

**DOI:** 10.1016/j.obpill.2025.100185

**Published:** 2025-06-06

**Authors:** Alejandro Campos, Kathryn L. Fantasia, Ivania Rizo

**Affiliations:** a Section of General Internal Medicine, Boston Medical Center, Boston, MA, United States; b Department of Medicine, Boston University Chobanian and Avedisian School of Medicine, Boston, MA, United States; c Evans Center for Implementation and Improvement Sciences, Department of Medicine, Boston University Chobanian and Avedisian School of Medicine, Boston, MA, United States; d Section of Endocrinology, Diabetes, Nutrition and Weight Management, Boston University Chobanian and Avedisian School of Medicine, Boston, MA, United States

## Abstract

**Background:**

Obesity is a highly prevalent, chronic, and treatable disease that disproportionately impacts some minoritized populations who seek care in safety-net settings. Given that primary care providers (PCPs) often serve as the initial point of contact for patients, we aimed to assess their knowledge, attitudes, and practices related to management of obesity.

**Methods:**

This was a cross-sectional study conducted to assess knowledge, attitudes, and practices on obesity management through an anonymous, electronic survey among trained (MD/DO and NP) and in-training (residents) primary care providers (PCPs) in the Departments of Internal Medicine and Family Medicine within an urban safety-net healthcare system.

**Results:**

Among 350 sampled, 96 PCPs completed the survey (27 % response rate). Participants were predominantly (60.4 %) Internal Medicine trainees. The majority of PCPs accurately identified common weight-related comorbidities and improvement of these with >10 % weight loss. Only 25 % of PCPs correctly identified both body mass index (BMI) criteria for anti-obesity medication (AOM) prescription and only 9.1 % identified both BMI criteria for bariatric surgery. Nearly two-thirds (64 %) of PCPs reported prescribing AOMs, with greater comfort in using glucagon like peptide-1 receptor agonist (GLP-1 RA) injectable agents (semaglutide and liraglutide) than with older oral AOMs (phentermine, phentermine-topiramate, and naltrexone-bupropion). Knowledge about side effects and insurance coverage were reported as influencing AOM prescription. Time constraints and lack of training and/or knowledge were identified as barriers in obesity management by more than 50 % of PCPs.

**Conclusions:**

Our study highlights gaps in obesity-related knowledge and practice among PCPs, emphasizing the need for enhanced training, clinical support, and policy reforms to improve obesity management and patient outcomes.

## Graphical abstract

Summary of a cross-sectional survey assessing knowledge, attitudes, and practices in obesity management among trained and in-training primary care providers at a large urban safety-net hospital. Findings highlight limited recognition of anti-obesity medication (AOM) prescribing criteria, moderate prescribing rates, and barriers such as time constraints and knowledge gaps. **Abbreviations:** MD, Doctor of Medicine; DO, Doctor of Osteopathic Medicine; NP, Nurse Practitioner; PCP, Primary Care Provider; AOM, Anti-Obesity Medication; GLP1, Glucagon-Like Peptide-1 receptor agonist; BMI, Body Mass Index.

**Figure:**
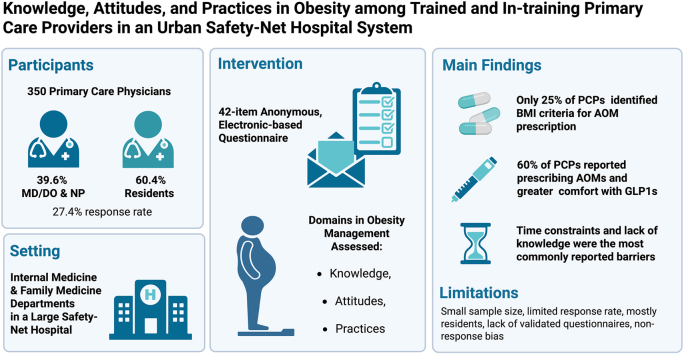

## 1 Introduction

Obesity is a highly prevalent, chronic, and treatable disease that poses significant challenges to both the medical community and society at large. Over the past thirty years, the global prevalence of obesity has doubled, affecting more than 890 million adults in 2022 and poses a massive economic burden [1]. Marked by high rates of mortality and morbidity, obesity is strongly linked to a wide range of comorbidities, most notably ischemic heart disease—the leading cause of death worldwide [2]. Obesity and its complications disproportionately affect individuals of all ages with lower socioeconomic status and from racial and ethnic minority communities, who are predominantly cared for in safety-net hospitals, community health centers, and public clinics [3,4]. These healthcare settings serve as critical access points for these populations facing structural barriers in obesity care, including lack of insurance, limited healthcare resources, and systemic inequities in obesity treatment [5,6].

Primary care providers (PCPs) play a central role in the early diagnosis and treatment of obesity; however, significant knowledge gaps, systemic biases, and structural barriers limit effective obesity care [7]. Multiple survey studies [[8], [9], [10], [11], [12], [13], [14], [15], [16], [17], [18], [19], [20], [21], [22], [23], [24], [25], [26], [27]] among PCPs have identified knowledge gaps in weight-related comorbidities, the benefits of weight loss, and the appropriate use of anti-obesity medications (AOM) or bariatric surgery referrals, structural barriers related to time constraints and insurance coverage, and significant bias in obesity care. These limitations contribute to suboptimal practices in prevention, diagnosis, and treatment, ultimately limiting effective obesity management. This, in turn, exacerbates both the public and individual burden of obesity. While previous research has identified knowledge gaps in obesity care, limited data exists on PCPs in safety-net settings, where obesity is more prevalent. Our study aimed to fill this gap in the literature by using a knowledge, attitudes, and practices questionnaire to assess trained and in-training PCPs at the largest urban safety-net hospital in New England.

## 2 Methods

### 2.1 Study design

This was a cross-sectional study conducted to assess knowledge, attitudes, and practices on obesity management through an anonymous, electronic survey among trained and in-training primary care providers (PCPs) in the Departments of Internal Medicine and Family Medicine within an urban safety-net healthcare system. This study was determined as exempt and approved by the Boston University Medical Campus and Boston Medical Center Institutional Board Review Board **(**H-43547). All respondents provided electronic consent.

### 2.2 Study setting

This study was conducted at Boston Medical Center (BMC), the largest urban, safety-net hospital in New England. A safety-net hospital is a healthcare facility that delivers care to all individuals regardless of insurance status or ability to pay, and often serves a disproportionately high number of Medicaid, uninsured, and socioeconomically disadvantaged patients. BMC serves a predominantly low-income, racially, and ethnically diverse population. More than 58 % of BMC's patients are enrolled in Medicaid, with approximately 32 % identifying as Black or African American and 24 % identifying as Hispanic/Latino [28,29]. A substantial portion of BMC's patient population is impacted by adverse social determinants of health, including housing instability and food insecurity, with an estimated 37 % of households living below the federal poverty line [30].

### 2.3 Study participants

The survey was distributed to both trained and in-training PCPs across internal medicine and family medicine practices at BMC. Trained PCPs included attending physicians (MD/DO) and nurse practitioners (NP). In-training PCPs included internal medicine and family medicine residents, excluding chief residents. At the time of distribution, the total pool included 350 PCPs across both departments.

### 2.4 Questionnaire design

The questionnaire was developed following a knowledge, attitudes, and practices survey model [31,32] and was adapted from existing questionnaires [26,33]. The questionnaire consisted of 43 items, divided into four sections: demographics, knowledge, attitudes, and practices. The demographics section included 7 items covering gender identity, age, professional role, specialty, level of training (post-graduate year), years in practice, and formal training on obesity. The knowledge section contained 11 items, including 7 multiple-choice questions, 2 open-ended questions, and 2 ranking/selection questions, designed to directly assess providers' knowledge. These questions covered topics such as the prevalence of obesity, body mass index (BMI) cutoffs for diagnosis, weight-related comorbidities, the effectiveness of various treatments, and the indications for using AOMs and bariatric surgery. The attitudes section included 9 items, all using a 5-point Likert scale, focusing on PCPs' beliefs about obesity, including their perception of obesity as a disease, its causes, and their confidence and level of comfort in managing it. Lastly, the practices section consisted of 16 items, including 10 frequency scale questions, 3 multiple-choice questions, and 3 open-ended questions. These assessed self-reported behaviors in clinical practice, such as how often providers diagnose obesity, prescribe anti-obesity medications, refer patients to weight management services, and the pragmatic barriers they face in treating obesity.

The questionnaire was reviewed by an expert in endocrinology and obesity medicine (IR) and an endocrinologist (KLF). Prior to distribution, the questionnaire was additionally reviewed by two internal medicine residents, two nurse practitioners, and two primary care physicians and was revised to improve clarity of the question-and-answer items and to limit length to less than 5 min. The finalized questionnaire is available in the Supplementary Material. The anonymous questionnaire was distributed from September to December 2023 via email with up to 3 automated reminders and administered via REDCap (Research Electronic Data Capture) tools hosted at Boston University [34].

### 2.5 Criteria for anti-obesity medications and bariatric surgery

We assessed participants’ knowledge of AOM prescription criteria and bariatric surgery indications using established guidelines. For AOM use, we defined appropriate prescription criteria based on BMI ≥27 kg/m^2^ with weight-related comorbidities or BMI ≥30 kg/m^2^ without comorbidities, consistent with current clinical guidelines at the time of the study [35]. For bariatric surgery, we used the 2022 American Society for Metabolic and Bariatric Surgery (ASMBS) criteria, which include BMI ≥30 kg/m^2^ with weight-related comorbidities or BMI ≥35 kg/m^2^ without comorbidities [36]. At the time of data collection, tirzepatide had not yet received FDA approval for weight loss, so it was not included in the questionnaire or study analysis.

### 2.6 Statistical analysis

Descriptive statistics are reported as frequency and percentage for discrete variables and as mean and standard deviation for continuous variables. Two-sample T tests and chi-square tests were used to compare baseline demographics and survey answers and to compare outcomes between level of training for participants who provided demographic information (n = 91 of 96). For the referral-related questions in the practices section, we dichotomized responses into “Frequent" (Always, Often) and “Infrequent" (Sometimes, Rarely, Never) to simplify analysis and better assess patterns of provider referrals. Survey data was analyzed using JMP Pro 17 and graphs were designed using GraphPad PRISM 10. A two-sided *p* value of less than 0.05 was considered statistically significant.

## 3 Results

### 3.1 Participant characteristics

A total of 96 of 350 sampled PCPs completed the knowledge, attitudes, and practices domains of the questionnaire, yielding a response rate of 27.4 %. Five participants did not provide demographic information. From the 91 respondents with demographic information, most were women between 20 and 30 years old. Sixty percent of the respondents were in-training PCPs, with nearly 90 % of those respondents being internal medicine residents. Participant characteristics are shown in Table 1.

**Table 1:** Demographics and baseline characteristics.

	N	*% of Total*
**Total Number of Respondents**	96^a^	*100 %*
**Self-reported Gender**		
Man	34	*37.4 %*
Non-Binary	1	*1.1 %*
Woman	56	*61.5 %*
**Age**		
20–30 years	45	*49.5 %*
31–40 years	25	*27.7 %*
41–50 years	9	*9.9 %*
51–60 years	6	*6.6 %*
>60 years	6	*6.6 %*
**Role**		
Attending MD/DO	27	*29.7 %*
Nurse Practitioner	9	*9.9 %*
Resident	55	*60.4 %*
**Level of Training**		
Trained PCP	36	*39.6 %*
In-training PCP	55	*60.4 %*
**Specialty**		
Family Medicine	7	*12.7 %*
Internal Medicine	48	*87.3 %*
**Postgraduate Year Level of Training**		
1	15	*27.3 %*
2	23	*41.8 %*
3	17	*30.9 %*
**Obesity Training**		
No	24	*26.4 %*
Yes	67	*73.6 %*
**Obesity Training during medical school or nursing school**		
No	46	*47.9 %*
Yes	50	*52.1 %*
**Obesity Training during residency**		
No	54	*56.3 %*
Yes	42	*43.8 %*

Abbreviations: PCP, primary care physician.

a 96 participants completed the knowledge, attitudes, and practices domains of the questionnaire; only 91 participants provided complete demographic information.

### 3.2 Knowledge

**Fig. 1:**
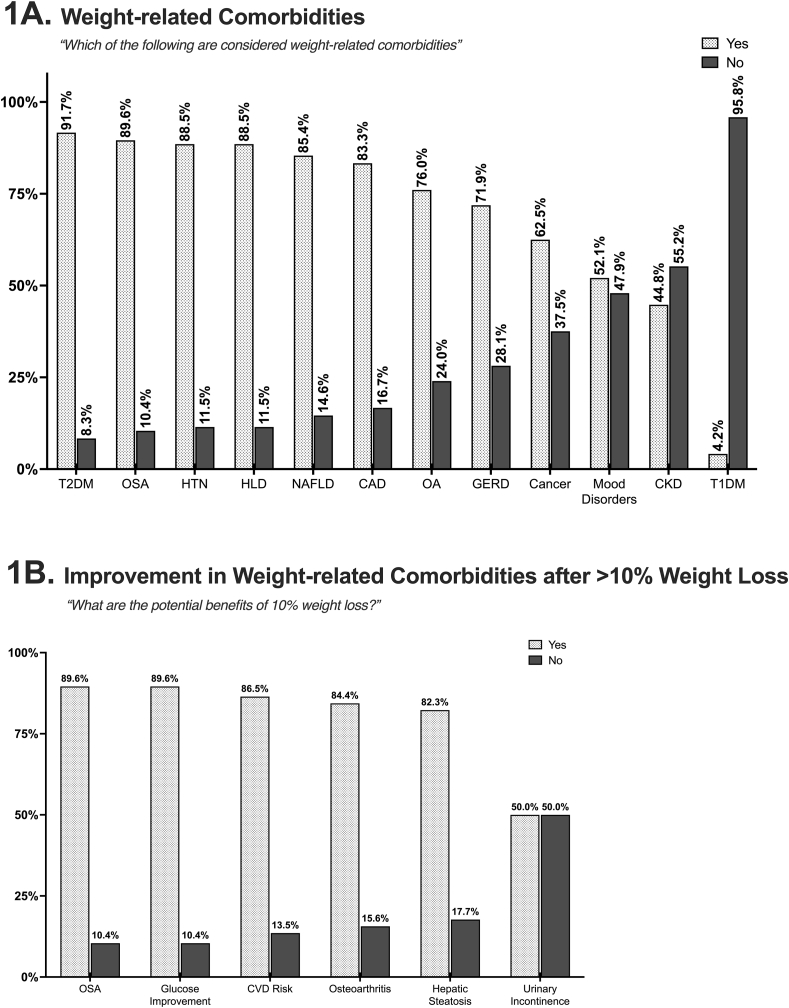
(A) Weight-related comorbidities identified by primary care providers (PCPs). (B) Comorbidities that PCPs recognize as improving with >10 % weight loss. The subtitle presents the survey questions displayed to participants. Abbreviations: T2DM, type 2 diabetes mellitus; OSA, obstructive sleep apnea; HTN, hypertension; HLD, hyperlipidemia; NAFLD, non-alcoholic fatty liver disease; CAD, coronary artery disease; OA, osteoarthritis; GERD, gastroesophageal reflux disease; CKD, chronic kidney disease; T1DM, type 1 diabetes mellitus; CVD, cardiovascular disease.

Among all respondents, approximately one quarter (26.4 %) reported no prior training in obesity medicine. Of those with prior training in obesity medicine, 52.1 % indicated receiving it during their medical or nursing education and 43.8 % during residency. Around 90 % correctly identified the BMI criteria for diagnosing obesity (≥30 kg/m^2^), however, 64.8 % were unaware of the lower BMI thresholds used for diagnosing obesity across different racial and ethnic groups, including individuals of Asian and South Asian ancestry. Although most respondents correctly recognized common weight-related comorbidities, fewer than 80 % identified cancer, gastro-esophageal reflux disease (GERD), osteoarthritis (OA), mood disorders, and chronic kidney disease (CKD) as obesity-related comorbidities (Fig. 1A).

Many PCPs identified bariatric surgery as the most effective and durable intervention to achieve >10 % weight loss (61.4 %), followed by diet (14.8 %), behavioral interventions (13.6 %), anti-obesity medications, and exercise. They also correctly identified the benefits of weight loss in managing conditions such as obstructive sleep apnea (OSA), metabolic-associated fatty liver disease (MASLD), OA, type 2 diabetes mellitus (T2DM), and reducing cardiovascular risk (Fig. 1B). Over 90 % correctly recognized that weight loss could lead to remission of T2DM. However, only half of the respondents considered weight loss beneficial for treating urinary incontinence. Notably, a higher percentage of trainees (83.6 %) compared to PCPs (61.1 %) perceived bariatric surgery as effective in reducing microvascular complications of T2D (p = 0.0253). Details on the analysis by level of training are presented in Supplementary Table S1A and S1B.

Regarding knowledge of BMI criteria for AOMs and bariatric surgery, only 27.1 % of respondents correctly identified BMI ≥27 with comorbidities as an indication for AOMs, and 46.9 % correctly recognized BMI ≥30 without comorbidities. Overall, just 25 % of respondents were able to correctly identify both BMI criteria for AOM use. For bariatric surgery, 20.8 % correctly identified BMI ≥30 with comorbidities as a criterion, while only 13.5 % correctly recognized BMI ≥35 without comorbidities. When considering both BMI criteria for bariatric surgery, only 9.4 % of respondents identified both correctly. There were no significant differences in these findings across levels of training.

### 3.3 Attitudes

A total of 87.5 % of PCPs considered obesity a disease. PCPs were surveyed regarding their preferences for terminology when referring to patients with obesity. Over 90 % expressed a preference for person-first language, such as “patients/people with obesity." Regarding beliefs about the causes of obesity, most PCPs disagreed that lack of exercise/physical activity or willpower were primary contributors, with 42.1 % rating willpower as “strongly disagree” and 33 % exercise/physical activity as “disagree”.

Overall, self-reported confidence in obesity management were generally neutral to slightly positive, with most PCPs selecting 'agree' or 'neither agree nor disagree,' while few responded with 'strongly agree' or shifted toward disagreement. In regard to confidence in overall obesity management, 37.5 % reported ‘agree’ and 35.2 % reported ‘neither agree nor disagree’. Most agreed that they were confident in providing dietary and nutritional advice (44.3 %) and guidance on exercise and physical activity (51.1 %) for weight management.

**Fig. 2:**
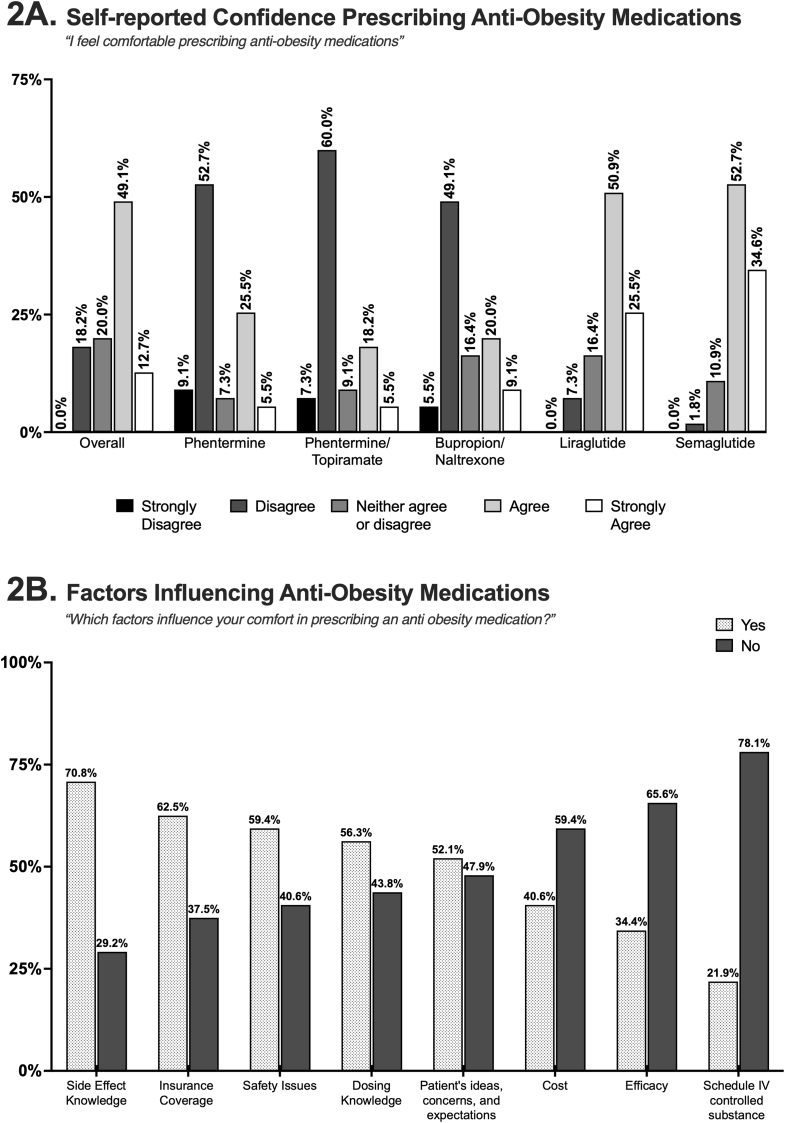
(A) Self-reported confidence among primary care providers (PCPs) in prescribing anti-obesity medications (AOMs), shown overall (left) and stratified by specific medications. (B) Factors that PCPs identify as influencing AOMs prescription. The subtitle presents the survey questions displayed to participants.

Almost half of PCPs (49.1 %) felt comfortable prescribing AOMs, with fewer than 20 % expressing discomfort. PCPs expressed the greatest comfort with GLP-1 analogues such as Liraglutide (Saxenda®) and Semaglutide (Wegovy®). In contrast, they reported less comfort with Phentermine, Phentermine-Topiramate (Qsymia®), and Bupropion-Naltrexone (Contrave®) (Fig. 2A). The self-reported level of comfort with prescription of phentermine (p = 0.0293) and liraglutide (p = 0.0184) was significantly lower among in-training PCPs. Key factors influencing comfort with AOMs included knowledge of dosing, awareness of side effects, safety concerns, insurance coverage, and beliefs about patient specific considerations such as ideas, concerns, and expectations (Fig. 2B). A notable difference was observed between trained PCPs and in training PCPs, with 72.7 % of trained PCPs considering patients' perspectives important compared to only 58.5 % of in-training PCPs (p = 0.01). Efficacy, cost, or the classification of some AOMs as schedule IV-controlled substances (e.g., phentermine) did not significantly impact comfort levels. Details on the analysis by level of training are presented in Supplementary Table S2A and S2B.

### 3.4 Practices

When surveyed about their practices in diagnosing obesity, under half of PCPs reported to “sometimes” (43.7 %) include obesity as a visit diagnosis for patients with a BMI ≥30 kg/m^2^. Analysis by training level revealed that trained PCPs were significantly more likely to consistently include obesity as a diagnosis compared to in-training PCPs (p = 0.0005).

Nearly two-thirds of the PCPs (64 %) reported prescribing AOMs. Referral responses were dichotomized into “Frequent" (Always, Often) and “Infrequent" (Sometimes, Rarely, Never) categories for analysis. Overall, 50.6 % of PCPs reported frequently referring patients to Obesity Medicine when appropriate, whereas only 17.2 % reported frequently referring patients for bariatric surgery.

**Fig. 3:**
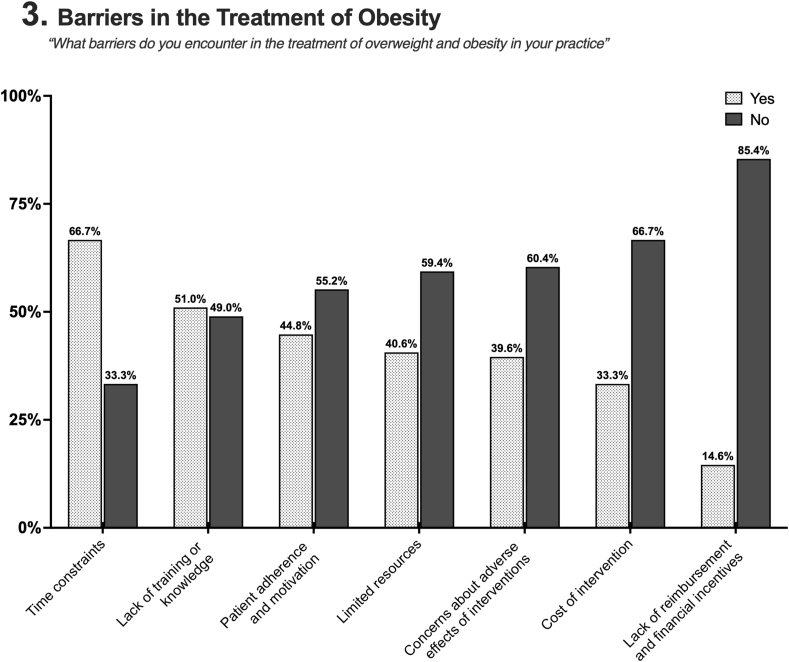
Barriers identified by PCPs in obesity management. The subtitle presents the survey question displayed to participants.

Most PCPs deemed referrals to Obesity Medicine specialists appropriate based on patient requests, BMI, presence of weight-related comorbidities, failure of lifestyle interventions, and inadequate response to AOMs. The primary barriers to obesity treatment reported included time constraints and a lack of training or knowledge. Limited resources, reimbursement issues, cost of interventions, concerns about adverse effects, and patient adherence were less frequently perceived as barriers (Fig. 3). Details on the analysis by level of training are presented in Supplementary Table S3.

## 4 Discussion

Our study identified persistent gaps in obesity-related knowledge, along with patterns of attitudes and practices among PCPs [[8], [9], [10], [11], [12], [13], [14], [15], [16], [17], [18], [19], [20], [21], [22], [23], [24], [25], [26], [27]]. To our knowledge, this is the first study to assess obesity-related knowledge, attitudes, and practices among both trained and in-training providers in a large, urban safety-net setting, spanning two key specialties in adult primary care. Notably, only 25 % of respondents correctly identified the BMI criteria for AOM use, and just 9.4 % correctly identified the ASMBS 2022 criteria for bariatric surgery eligibility, suggesting that these knowledge deficits likely contribute to the underutilization of evidence-based interventions. Although most PCPs acknowledged obesity as a disease and largely moved away from attributing it solely to lifestyle factors, greater proportions of respondents referred to diet and exercise than to comprehensive obesity medicine clinics or surgical options, potentially reflecting an entrenched perception shaped by limited clinical exposure and training in obesity management.

Over 80 % of respondents correctly identified common obesity-related comorbidities such as T2DM and cardiovascular disease; however, associations with CKD, GERD, OA, mood disorders, and certain cancers were less frequently recognized. Addressing these awareness gaps may heighten the urgency of treating obesity and encourage a more comprehensive approach that recognizes obesity as a primary driver of comorbidities, prioritizing its management to improve patient outcomes rather than solely treating comorbidities in isolation. Furthermore, while over 60 % of PCPs reported prescribing AOMs—with a preference for newer injectable agents (GLP-1 analogues)—a substantial proportion expressed discomfort regarding side effect profiles and insurance-related barriers. A little over 60 % of PCPs identified bariatric surgery as the most effective and durable intervention to achieve >10 % weight loss, however, only 17 % of them reported frequently referring patients to bariatric surgery. Finally, the most reported barrier to treatment of obesity was a lack of time, suggesting that additional support for PCPs within the primary care environment may be beneficial. Implementing primary care embedded obesity visits and group-based and virtual care models for weight management offers a promising approach to effectively address obesity within the primary care setting [7]. These findings highlight an urgent need for enhanced training across all levels of medical education, as well as streamlined clinical processes and policy-level interventions to reduce administrative burdens and support PCPs in delivering obesity care.

Future interventions across multiple levels of the socio-ecological model of health should be considered to comprehensively address obesity management. At the individual level, targeted educational programs integrated into graduate, postgraduate, and continuing medical education could enhance clinician competence and confidence in prescribing AOMs and making appropriate referrals for bariatric surgery. Olson et al. showed that implementing a preclinical curriculum in obesity improved attitudes and knowledge among medical students [37]. Similarly, Chae et al. demonstrated that a six-month multicomponent obesity curriculum in an Internal Medicine residency enhanced skills in nutrition, behavior modification, and obesity-related pharmacotherapy and surgical counseling [38]. Interpersonally, mentorship programs and peer-support networks may facilitate knowledge transfer between trained and in-training providers, fostering a collaborative learning environment. Embedded obesity management clinics within primary care settings with supportive staff roles (e.g., nurses, MAs) could alleviate time constraints by centralizing specialized services [7]. These embedded clinics could also serve as hubs for the dissemination of best practices and potentially creating obesity champions in primary care. Such ‘condition-specific’ embedded clinics have been demonstrated to be effective in increasing the treatment of Hepatitis C and management of opioid use disorder in primary care [[39], [40], [41]]. Champions can also serve as designated change agents-driving policy initiatives, standardizing best practices, and fostering a supportive environment for obesity management through mentoring, collaboration, and facilitation of knowledge sharing [42]. At the community level, partnerships with local organizations and public health departments could enhance patient engagement and adherence through culturally tailored interventions, thereby bridging the gap between clinical care and community resources. Finally, policy-level initiatives, such as advocating for expanded insurance coverage for AOMs, streamlined prior authorization processes, and encouraging government negotiation or regulation to lower medication prices are essential to dismantle systemic barriers and promote equitable access to care. By addressing obesity management through these multi-level strategies, future interventions have the potential to significantly improve patient outcomes in high-burden, safety-net populations.

### 4.1 Strengths and limitations

Strengths of our study include its setting within the largest safety-net hospital system in New England, allowing us to assess clinicians treating a diverse patient population disproportionately affected by obesity. Further, our study involved both trained and in-training PCPs from Internal Medicine and Family Medicine departments. While 60 % of respondents were in-training PCPs, findings did not significantly differ by level of training. Further post hoc analyses stratified by specialty were limited by the overall sample size, modest response rate, and the low proportion of family medicine respondents, who comprised only 13 % of the sample. However, the primary aim of the study was to assess PCPs as a whole rather than to compare differences between specialties or by training level. Despite its strengths in capturing data from a relevant and underrepresented population in healthcare research, the study has several limitations. First, it is a single-center study, which limits generalizability to other settings. However, as the study included PCPs across multiple disciplines (internal medicine and family medicine), and patterns were similar to those in non-safety-net settings, findings may be transferrable to clinicians who practice in other safety-net settings. Another important limitation of our study is the response rate, which was 27 %. However, prior studies have reported highly variable sample sizes (ranging from 100 to 1944) and response rates (from 18.5 % to 92 %) [[8], [9], [10], [11], [12], [13], [14], [15], [16], [17], [18], [19], [20], [21], [22], [23], [24], [25], [26], [27]], and our response rate was consistent with other studies using email-based survey methods [[8], [9], [10]]. Still, the modest response rate in our study raises the possibility of non-response bias, particularly due to lower participation among trained PCPs. Additionally, the predominance of in-training respondents may have influenced the findings. This may limit the representativeness of the sample and the generalizability of the findings to the broader population of PCPs in safety-net settings. Future studies should consider approaches to enhance engagement, such as offering incentives or incorporating the survey into educational or training initiatives. While our questionnaire was adapted from validated instruments and reviewed by experts, we did not formally assess its psychometric properties in assessing attitudes and practices. However, we did model our questionnaire after those that have been previously used and on an established knowledge, attitudes, and practices questionnaire model. Lastly, while anonymity helped protect participant privacy, it also limited our ability to systematically assess non-respondent bias.

### 4.2 Conclusions

The observed deficits in obesity-related knowledge, attitudes, and practices among PCPs underscore the urgent need for multifaceted interventions —spanning medical education, clinical infrastructure improvements, and systemic policy reforms. While our findings should be interpreted in light of the study's methodological limitations, they emphasize a critical opportunity to strengthen obesity care in safety-net settings, where the burden is greatest and a “treat obesity first” approach could markedly improve patient outcomes.

### 4.3 Key takeaways

1. Persistent Knowledge Gaps

Only 25 % of PCPs correctly identified the BMI criteria for AOMs use, and fewer than 10 % identified both BMI criteria for bariatric surgery.

2. Confidence and Comfort Vary by Medication Type and Training Level

Nearly two-thirds of PCPs reported prescribing AOMs, and comfort was significantly higher with GLP-1 receptor agonists than with older oral agents.

3. Structural and Educational Barriers Impact Care

Time constraints and lack of training or knowledge were the most reported barriers to obesity management.

## Ethics approval

This study was approved by the Boston University Medical Campus and Boston Medical Canter Institutional Board Review Board (H-43547).

## Consent to participate declaration

All participants provided electronic consent.

## Declaration consent for publication declaration

Not applicable.

## Availability of data sharing and transparency

Data collected for the study, including individual de-identified participant data, will be available to interested parties after the signing of a data access agreement. Data may be requested by contacting the corresponding author.

## Disclosures

AC as an editor on the Obesity Pillars board.

## Artificial intelligence use statement

Artificial intelligence tools were used to assist with grammar and spelling correction during manuscript preparation. No AI tools were used for data analysis, interpretation, or content generation.

## Licenses

Graphical Abstract created in BioRender. Campos, A. (2025) https://BioRender.com/m16ejyi.

## Author contribution

All authors had full access to all the data and statistical analyses.

IR had full access to all the data in the study and takes responsibility for the integrity of the data and the accuracy of the data analysis.

*Concept and design*: AC, KF, IR.

*Acquisition, analysis, or* interpretation of data: AC, KF, IR.

Drafting of the manuscript: AC, KF, IR.

*Critical revision of the manuscript for important intellectual content:* AC, KF, IR.

*Statistical analysis:* AC, KF, IR; *Administrative, technical, or material* support*:* AC, KF, IR.

## Clinical trial registration

None.

## Funding:

Effort on this manuscript was made possible for 10.13039/501100016148Kathryn L. Fantasia by K12DK133995, USA NIH NIDDK grant.

No funding sources were involved in the study design, data collection, analysis, or interpretation, manuscript drafting and writing, or in the decision to submit the manuscript for publication.
