# Supplementary material for: Knowledge, attitudes, and practices in obesity among trained and in-training primary care providers in an urban safety-net hospital system
Source: Obes Pillars. 2025 Jun 6;15:100185. doi: 10.1016/j.obpill.2025.100185 (PMC12182768; doi:10.1016/j.obpill.2025.100185)
Supplement: Multimedia component 1 [file mmc1.pdf]

Knowledge, Attitudes, and Practice in Obesity

You are being asked to voluntarily participate in an anonymous survey to assess the attitudes, knowledge and practices in obesity among primary care providers at Boston Medical Center. If you agree, you will be directed to A brief 5 to 10 minutes survey.If you have any questions, please email aacampos@bu.edu

I am willing to complete this questionnaire	◯ Yes<br>◯ No
Gender identity	◯ Man<br>◯ Woman<br>◯ Non-binary/non-conforming<br>◯ Gender Queer<br>◯ Prefer not to answer<br>◯ Preferred to self describe as
If selected preferred to self describe as, please describe in a few words	______________________
Age	◯ 20 to 30 years<br>◯ 31 to 40 years<br>◯ 41 to 50 years<br>◯ 51 to 60 years<br>◯ >60 years
Professional role	◯ Attending physician<br>◯ Nurse practitioner<br>◯ Medical Resident
Specialty	◯ Internal Medicine<br>◯ Family Medicine
PGY Level	◯ 1<br>◯ 2<br>◯ 3<br>◯ 4 (Chief Resident)
How many years have you been in practice?	◯ 0 to 5 years<br>◯ 5 to 10 years<br>◯ 11 to 20 years<br>◯ 21 to 30 years<br>◯ >30 years
During your training (medical school, nursing school, residency) did you receive formal training on obesity? (e.g., module, grand rounds, elective)	◯ Yes<br>◯ No
Please select one or more options of when you received your training	☐ Nursing/Medical School<br>☐ Residency.
I think Obesity is a disease	◯ Yes<br>◯ No
What is the prevalence (%) of obesity (BMI >30 kg/m2) in 2022 in the USA?	______________________<br>(%)

In adults, what is the BMI cutoff for obesity?	◯ ≥20<br>◯ ≥25<br>◯ ≥30<br>◯ ≥35<br>◯ ≥40
Is there a difference in estimating obesity by BMI cutoffs based on race or ethnicity?	◯ Yes<br>◯ No
Which of the following are considered weight-related comorbidities	☐ Type 1 diabetes<br>☐ Type 2 diabetes<br>☐ NAFLD<br>☐ Dyslipidemia<br>☐ Hypertension<br>☐ Obstructive Sleep Apnea<br>☐ Coronary Artery Disease<br>☐ Cancer<br>☐ Gastroesophageal Reflux Disease<br>☐ Osteoarthritis<br>☐ Mood disorders<br>☐ Chronic Kidney Disease<br>(Please select all that apply)
Weight loss alone can induce type 2 diabetes mellitus remission?	◯ Yes<br>◯ No
What is the most effective and durable intervention/treatment for >10% weight loss?	◯ Diet<br>◯ Exercise<br>◯ Behavioral interventions<br>◯ Pharmacotherapy<br>◯ Bariatric surgery
What are the potential benefits of 10% weight loss?	☐ Apnea Hypopnea Index Improvement in Obstructive Sleep Apnea<br>☐ Hepatic Steatosis reduction<br>☐ Urinary incontinence improvement<br>☐ Knee pain and function in persons with osteoarthritis<br>☐ Glycemic improvement<br>☐ Cardiovascular risk reduction<br>(Please select all that apply)
What are the indications for using anti-obesity medications?	☐ BMI ≥25 with weight-related comorbidity<br>☐ BMI ≥27 with weight-related comorbidity<br>☐ BMI ≥30 with weight-related comorbidity<br>☐ BMI ≥25 without weight-related comorbidity<br>☐ BMI ≥27 without weight-related comorbidity<br>☐ BMI ≥30 without weight-related comorbidity<br>☐ No BMI cutoff needed, indicated if provider considers appropriate for patient.<br>(Please select all that apply)

What are the current indications for bariatric surgery in adults?	☐ BMI 30-34.9 with weight-related comorbidity<br>☐ BMI ≥35 with weight-related comorbidity<br>☐ BMI ≥40 with weight-related comorbidity<br>☐ BMI 30-34.9 without weight-related comorbidity<br>☐ BMI ≥35 without weight-related comorbidity<br>☐ BMI ≥40 without weight-related comorbidity<br>☐ No BMI cutoff needed, indicated if provider considers appropriate for patient.<br>(Please select all that apply)
Potential Bariatric surgery outcomes include which of the following:	☐ Type 2 diabetes mellitus remission<br>☐ Decrease incidence of diabetes microvascular complications<br>☐ Lower risk of obesity-associated cancer<br>☐ Reduced incidence of heart failure, myocardial infarction, and stroke<br>☐ Esthetic Outcomes<br>(Please select all that apply)
What is the 30-day mortality rate for bariatric surgery?	◯ < 0.1%<br>◯ < 1%<br>◯ 2-5%<br>◯ 5-10%
In your opinion, the recommended terminology is:	◯ Obese patients/people<br>◯ Patients/people with obesity<br>◯ Both are recommended
In your opinion, obesity is caused by lack of will power	◯ Strongly agree<br>◯ Agree<br>◯ Neither agree nor disagree<br>◯ Disagree<br>◯ Strongly disagree
In your opinion, obesity is caused by lack of exercise/physical activity	◯ Strongly agree<br>◯ Agree<br>◯ Neither agree nor disagree<br>◯ Disagree<br>◯ Strongly disagree
I feel confident managing obesity	◯ Strongly agree<br>◯ Agree<br>◯ Neither agree nor disagree<br>◯ Disagree<br>◯ Strongly disagree
I feel confident providing diet and nutritional advice for weight management in obesity	◯ Strongly agree<br>◯ Agree<br>◯ Neither agree nor disagree<br>◯ Disagree<br>◯ Strongly disagree
I feel confident providing exercise/physical activity advice for weight management in obesity	◯ Strongly agree<br>◯ Agree<br>◯ Neither agree nor disagree<br>◯ Disagree<br>◯ Strongly disagree

When a patient has a BMI ≥30, I include Obesity as a visit diagnosis:	◯ Always<br>◯ Most of the times<br>◯ Sometimes<br>◯ Rarely<br>◯ Never
Do you prescribe anti-obesity medications (AOMs)?	◯ Yes<br>◯ No
What percentage of patients who meet criteria for anti obesity medication, do you prescribe an anti obesity medication?	______________________________<br>(%)
Do you feel comfortable prescribing anti obesity medications?	◯ Strongly agree<br>◯ Agree<br>◯ Neither agree nor disagree<br>◯ Disagree<br>◯ Strongly disagree
I feel comfortable prescribing Phentermine (ADIPEX-P, IONAMIN, PRO-FAST, etc)	☐ Strongly agree<br>☐ Agree<br>☐ Neither agree nor disagree<br>☐ Disagree<br>☐ Strongly disagree
I feel comfortable prescribing Phentermine/Topiramate (QSYMIA)	☐ Strongly agree<br>☐ Agree<br>☐ Neither agree nor disagree<br>☐ Disagree<br>☐ Strongly disagree
I feel comfortable prescribing Naltrexone/Bupropion (CONTRAVE)	☐ Strongly agree<br>☐ Agree<br>☐ Neither agree nor disagree<br>☐ Disagree<br>☐ Strongly disagree
I feel comfortable prescribing Liraglutide (SAXENDA)	☐ Strongly agree<br>☐ Agree<br>☐ Neither agree nor disagree<br>☐ Disagree<br>☐ Strongly disagree
I feel comfortable prescribing Semaglutide (WEGOVY)	☐ Strongly agree<br>☐ Agree<br>☐ Neither agree nor disagree<br>☐ Disagree<br>☐ Strongly disagree

Which factors influence your comfort in prescribing an anti obesity medication?	☐ Patient's ideas, concerns, and expectations<br>☐ Knowledge about dosing<br>☐ Knowledge about side effects<br>☐ Safety Issues<br>☐ Efficacy<br>☐ Cost<br>☐ Insurance Coverage<br>☐ Schedule IV controlled substance (i.e., phentermine)<br>☐ Other<br>(Please select all that apply)
If selected other, please describe in a few words	______________________________
When appropriate or indicated, how often do you provide exercise prescriptions?	◯ Never<br>◯ Rarely<br>◯ Sometimes<br>◯ Often<br>◯ Always
When appropriate or indicated, how often do you refer to nutrition or dietitian services?	◯ Never<br>◯ Rarely<br>◯ Sometimes<br>◯ Often<br>◯ Always
When appropriate or indicated, how often do you refer to medical weight management or obesity medicine?	◯ Never<br>◯ Rarely<br>◯ Sometimes<br>◯ Often<br>◯ Always
When appropriate or indicated, how often do you refer to bariatric surgery?	◯ Never<br>◯ Rarely<br>◯ Sometimes<br>◯ Often<br>◯ Always
When do you feel it is appropriate to refer to obesity medicine	☐ Patient request<br>☐ BMI<br>☐ Presence of weight-related comorbidity(ies)<br>☐ Lifestyle intervention(s) failure<br>☐ Antiobesity Medication(s) failure<br>☐ Request/needed by another specialist taking care of patient<br>☐ Other<br>(Please select all that apply)
If selected other, please describe in a few words	______________________________

What barriers do you encounter in the treatment of overweight and obesity in your practice	☐ Lack of training or knowledge<br>☐ Time constraints<br>☐ Limited resources<br>☐ Lack of reimbursement and financial incentives<br>☐ Cost of intervention<br>☐ Concerns about adverse effects of interventions<br>☐ Patient adherence and motivation<br>☐ Other<br>(Please select all that apply)
If selected other, please describe in a few words	__________________________
Please provide any feedback or questions about the questionnaire:	________________________________<br>(Optional)
