# Supplementary material for: Knowledge, attitudes, and practices in obesity among trained and in-training primary care providers in an urban safety-net hospital system
Source: Obes Pillars. 2025 Jun 6;15:100185. doi: 10.1016/j.obpill.2025.100185 (PMC12182768; doi:10.1016/j.obpill.2025.100185)
Supplement: Multimedia component 2 [file mmc2.docx]

| **Table S1A. Which of the following are considered weight-related comorbidities** | | | | | | | |
| --- | --- | --- | --- | --- | --- | --- | --- |
|  | **All PCPs** | | **Trained (MD/DO, NP)** | | **In-Training (Residents)** | | **P value for difference** |
| **T1DM** | | | | | | | |
| No | 92 | 95.83% | 35 | 97.22% | 52 | 94.55% | ***1.00000*** |
| Yes | 4 | 4.17% | 1 | 2.78% | 3 | 5.45% |  |
| **T2DM** | | | | | | | |
| No | 8 | 8.33% | 2 | 5.56% | 1 | 1.82% | ***0.55990*** |
| Yes | 88 | 91.67% | 34 | 94.44% | 54 | 98.18% |  |
| **NAFLD** | | | | | | | |
| No | 14 | 14.58% | 5 | 13.89% | 4 | 7.27% | ***0.47450*** |
| Yes | 82 | 85.42% | 31 | 86.11% | 51 | 92.73% |  |
| **HLD** | | | | | | | |
| No | 11 | 11.46% | 4 | 11.11% | 2 | 3.64% | ***0.20880*** |
| Yes | 85 | 88.54% | 32 | 88.89% | 53 | 96.36% |  |
| **HTN** | | | | | | | |
| No | 11 | 11.46% | 3 | 8.33% | 3 | 5.45% | ***0.67770*** |
| Yes | 85 | 88.54% | 33 | 91.67% | 52 | 94.55% |  |
| **OSA** | | | | | | | |
| No | 10 | 10.42% | 4 | 11.11% | 1 | 1.82% | ***0.07780*** |
| Yes | 86 | 89.58% | 32 | 88.89% | 54 | 98.18% |  |
| **CAD** | | | | | | | |
| No | 16 | 16.67% | 3 | 8.33% | 8 | 14.55% | ***0.51650*** |
| Yes | 80 | 83.33% | 33 | 91.67% | 47 | 85.45% |  |
| **Cancer** | | | | | | | |
| No | 36 | 37.50% | 8 | 22.22% | 23 | 41.82% | ***0.07100*** |
| Yes | 60 | 62.50% | 28 | 77.78% | 32 | 58.18% |  |
| **GERD** | | | | | | | |
| No | 27 | 28.13% | 6 | 16.67% | 16 | 29.09% | ***0.21600*** |
| Yes | 69 | 71.88% | 30 | 83.33% | 39 | 70.91% |  |
| **OA** | | | | | | | |
| No | 23 | 23.96% | 5 | 13.89% | 13 | 23.64% | ***0.29360*** |
| Yes | 73 | 76.04% | 31 | 86.11% | 42 | 76.36% |  |
| **Mood Disorders** | | | | | | | |
| No | 46 | 47.92% | 14 | 38.89% | 27 | 49.09% | ***0.39260*** |
| Yes | 50 | 52.08% | 22 | 61.11% | 28 | 50.91% |  |
| **CKD** | | | | | | | |
| No | 53 | 55.21% | 21 | 58.33% | 27 | 49.09% | ***0.40110*** |
| Yes | 43 | 44.79% | 15 | 41.67% | 28 | 50.91% |  |

| **Table S1A. What are the potential benefits of 10% weight loss?** | | | | | | | |
| --- | --- | --- | --- | --- | --- | --- | --- |
|  | **All PCPs** | | **Trained (MD/DO, NP)** | | **In-Training (Residents)** | | **P value for difference** |
| **OSA** | | | | | | | |
| No | 10 | 10.42% | 3 | 8.33% | 2 | 3.64% | ***0.38060*** |
| Yes | 86 | 89.58% | 33 | 91.67% | 53 | 96.36% |  |
| **Hepatic Steatosis** | | | | | | | |
| No | 17 | 17.71% | 5 | 13.89% | 7 | 12.73% | ***1.0000*** |
| Yes | 79 | 82.29% | 31 | 86.11% | 48 | 87.27% |  |
| **Urinary Incontinence** | | | | | | | |
| No | 48 | 50.00% | 18 | 50.00% | 25 | 45.45% | ***0.83020*** |
| Yes | 48 | 50.00% | 18 | 50.00% | 30 | 54.55% |  |
| **Osteoarthritis** | | | | | | | |
| No | 15 | 15.63% | 5 | 13.89% | 5 | 9.09% | ***0.50890*** |
| Yes | 81 | 84.38% | 31 | 86.11% | 50 | 90.91% |  |
| **Glucose Improvement** | | | | | | | |
| No | 10 | 10.42% | 3 | 8.33% | 2 | 3.64% | ***0.380060*** |
| Yes | 86 | 89.58% | 33 | 91.67% | 53 | 96.36% |  |
| **CVD Risk Improvement** | | | | | | | |
| No | 13 | 13.54% | 5 | 13.89% | 3 | 5.45% | ***0.25650*** |
| Yes | 83 | 86.46% | 31 | 86.11% | 52 | 94.55% |  |

| **Table S2A. I feel comfortable prescribing anti-obesity medications** | | | | | | | |
| --- | --- | --- | --- | --- | --- | --- | --- |
|  | **All PCPs** | | **Trained (MD/DO, NP)** | | **In-Training (Residents)** | | **P value for difference** |
| **Strongly Disagree** | 0 | 0.0% | 0 | 0.0% | 0 | 0.0% | ***0.5796*** |
| **Disagree** | 10 | 18.18% | 4 | 16.67% | 6 | 19.35% |  |
| **Neither agree nor disagree** | 11 | 20.00% | 3 | 12.50% | 8 | 25.81% |  |
| **Agree** | 27 | 49.09% | 13 | 54.17% | 14 | 45.16% |  |
| **Strongly agree** | 7 | 12.73% | 4 | 16.67% | 3 | 9.68% |  |

| **Table S2B. Which factors influence your comfort in prescribing an anti-obesity medication?** | | | | | | | |
| --- | --- | --- | --- | --- | --- | --- | --- |
|  | **All PCPs** | | **Trained (MD/DO, NP)** | | **In-Training (Residents)** | | **P value for difference** |
| **Patient's ideas, concerns, and expectations** | | | | | | | |
| No | 46 | 47.92% | 10 | 27.78% | 31 | 56.36% | ***0.0097*** |
| Yes | 50 | 52.08% | 26 | 72.22% | 24 | 43.64% |  |
| **Knowledge about dosing** | | | | | | | |
| No | 42 | 43.75% | 15 | 41.67% | 22 | 40.00% | ***1.0000*** |
| Yes | 54 | 56.25% | 21 | 58.33% | 33 | 60.00% |  |
| **Knowledge about side effects** | | | | | | | |
| No | 28 | 29.17% | 7 | 19.44% | 16 | 29.09% | ***0.3350*** |
| Yes | 68 | 70.83% | 29 | 80.56% | 39 | 70.91% |  |
| **Safety Issues** | | | | | | | |
| No | 39 | 40.63% | 11 | 30.56% | 23 | 41.82% | ***0.3758*** |
| Yes | 57 | 59.38% | 25 | 69.44% | 32 | 58.18% |  |
| **Efficacy** | | | | | | | |
| No | 63 | 65.63% | 25 | 69.44% | 33 | 60.00% | ***0.3829*** |
| Yes | 33 | 34.38% | 11 | 30.56% | 22 | 40.00% |  |
| **Cost** | | | | | | | |
| No | 57 | 59.38% | 18 | 50.00% | 34 | 61.82% | ***0.2866*** |
| Yes | 39 | 40.63% | 18 | 50.00% | 21 | 38.18% |  |
| **Insurance Coverage** | | | | | | | |
| No | 36 | 37.50% | 10 | 27.78% | 21 | 38.18% | ***0.3691*** |
| Yes | 60 | 62.50% | 26 | 72.22% | 34 | 61.82% |  |
| **Schedule IV controlled substance (i.e., phentermine)** | | | | | | | |
| No | 75 | 78.13% | 26 | 72.22% | 44 | 80.00% | ***0.4498*** |
| Yes | 21 | 21.88% | 10 | 27.78% | 11 | 20.00% |  |

| **Table S3. What barriers do you encounter in the treatment of overweight and obesity in your practice?** | | | | | | | |
| --- | --- | --- | --- | --- | --- | --- | --- |
|  | **All PCPs** | | **Trained (MD/DO, NP)** | | **In-Training (Residents)** | | **P value for difference** |
| **Lack of training or knowledge** | | | | | | | |
| No | 47 | 48.96% | 18 | 50.00% | 24 | 43.64% | ***0.6678*** |
| Yes | 49 | 51.04% | 18 | 50.00% | 31 | 56.36% |  |
| **Time constraints** | | | | | | | |
| No | 32 | 33.33% | 11 | 30.56% | 16 | 29.09% | ***1.0000*** |
| Yes | 64 | 66.67% | 25 | 69.44% | 39 | 70.91% |  |
| **Limited resources** | | | | | | | |
| No | 57 | 59.38% | 20 | 55.56% | 32 | 58.18% | ***0.8314*** |
| Yes | 39 | 40.63% | 16 | 44.44% | 23 | 41.82% |  |
| **Lack of reimbursement and financial incentives** | | | | | | | |
| No | 82 | 85.42% | 27 | 75.00% | 50 | 90.91% | ***0.0716*** |
| Yes | 14 | 14.58% | 9 | 25.00% | 5 | 9.09% |  |
| **Cost of intervention** | | | | | | | |
| No | 64 | 66.67% | 20 | 55.56% | 39 | 70.91% | ***0.1784*** |
| Yes | 32 | 33.33% | 16 | 44.44% | 16 | 29.09% |  |
| **Concerns about adverse effects of interventions** | | | | | | | |
| No | 58 | 60.42% | 19 | 52.78% | 34 | 61.82% | ***0.5147*** |
| Yes | 38 | 39.58% | 17 | 47.22% | 21 | 38.18% |  |
| **Patient adherence and motivation** | | | | | | | |
| No | 53 | 55.21% | 18 | 50.00% | 30 | 54.55% | ***0.8302*** |
| Yes | 43 | 44.79% | 18 | 50.00% | 25 | 45.45% |  |
